# Identification of novel stem cell markers using gap analysis of gene expression data

**DOI:** 10.1186/gb-2007-8-9-r193

**Published:** 2007-09-17

**Authors:** Paul M Krzyzanowski, Miguel A Andrade-Navarro

**Affiliations:** 1 Molecular Medicine, Ottawa Health Research Institute, 501 Smyth Road, Ottawa, Ontario, K1H 8L6, Canada; 2 Faculty of Medicine, University of Ottawa, 451 Smyth Road, Ottawa, Ontario, K1H 8M5, Canada

## Abstract

We describe a method for detecting marker genes in large heterogeneous collections of gene expression data. Markers are identified and characterized by the existence of demarcations in their expression values across the whole dataset, which suggest the presence of groupings of samples. We apply this method to DNA microarray data generated from 83 mouse stem cell related samples and describe 426 selected markers associated with differentiation to establish principles of stem cell evolution.

## Abstract 2

A method for the detection of marker genes in large heterogeneous collections of gene expression data is described and applied to DNA microarray data generated from 83 mouse stem cell-related samples.

## Background

Gene expression microarrays allow thousands of transcripts in a cellular sample to be quantified simultaneously. (For reviews of the technology and applications, see the reports by Heller [1] and Sloughton [2].) Continuing improvements in microarray technology, in terms of transcript density, technical robustness, and cost, have led to widespread usage of arrays in experiments. The size of single studies has grown and can encompass the analysis of up to hundreds of arrays simultaneously [3-5]. This vast explosion of reusable data being generated has resulted in efforts being directed at producing expression data repositories in which the data are curated and presented in an ordered manner [6-8]. The large number of data points makes such resources an exceptional source of biologic information.

Some common uses of gene expression data are the identification of co-regulated genes across many samples [9], identification of differentially expressed genes in samples of interest [10], and, more recently, analysis of alternative splicing [11-13] and genome-wide surveillance of transcription [14-16]. They can also be used to identify marker genes associated with specific sets of samples. As distinguishing features, such markers can be used as diagnostic tests for disease [17,18] or for the identification and purification of particular cell types [19,20]. The identification of multiple markers for a particular phenotype may also reveal biologic mechanisms by which certain genes act in concert.

A simple method to identify marker gene candidates is to identify genes that are differentially expressed between a set of control samples and samples from a condition of interest. A two-state comparison can be made, and genes associated with each type of sample can be identified and used as markers. Current gene expression databases typically contain data from many types of samples, and this heterogeneity provides the potential for more powerful analyses. One can, for example, identify transcripts that are specific to a sample (or samples) of interest, or conduct novel comparisons between different combinations of transcription profiles. The increased size of the databases also increases the number of possible two-state comparisons exponentially, which poses a computational problem. Overcoming this problem requires a computational method.

We have developed a methodology that uses large heterogeneous gene expression datasets to identify genes that can function as markers. In summary, we examine the distribution of expression values of each probe set to identify gaps. These gaps can be used to partition the database into groups of low-expressing and high-expressing samples, which suggest the existence of distinct subpopulations of samples. We then score other probe sets based on their ability to reproduce these database partitions. The characteristics of samples in each database partition identify the context in which genes may act as markers, which aids in the subsequent evaluation of genes in terms of their putative marker roles.

In this study we illustrate our methodology in the analysis of a database of stem-cell related DNA microarray samples that we previously developed (StemBase [7]). In particular, we study 83 mouse stem cell related samples analyzed using the Affymetrix MOE430 genechip set (Affymetrix Inc., Santa Clara, CA, USA), which includes approximately 45,000 probe sets. Unbiased application of the method produces a set of 4,449 cell and tissue markers, including 45 out of 71 known stem cell markers (69%). Analysis of the markers that segregate six types of stem cells (hematopoietic, mast, mammospheres, osteoblasts, and two embryonic) from their differentiated counterparts suggests 426 high confidence markers, 206 of which are highly expressed in the stem cell and 222 are highly expressed in the differentiated counterpart (two being highly expressed in stem cells in some cases, and in the differentiated counterpart in others). Of those 426 markers, 17 are involved in multiple distinct lineages that include at least one non-embryonic cell type; nine markers are highly expressed in the stem cells, six are highly expressed in the differentiated cells, and two exhibit opposite variation in different stem-derivative cell pairs. Analysis of the functions of the 222 genes that are highly expressed in the differentiated cells indicates enrichment of extracellular gene products and enzyme inhibitors (12 genes, five of them serpins).

The set of 426 stem cell markers allows us to focus on gene superfamilies that have undergone repeated gene duplication events for a phylogenetic analysis of the evolution of proteins involved in stem cell function. By sequence similarity analysis, we identify four such families (nuclear receptors, cytochrome P450, Rab family GTPases, and early B-cell factors) with multiple members in this set. The study of examples from each reveals multiple events of gene duplication along the vertebrate lineage giving rise to genes with a very high degree of sequence similarity, but very different patterns of expression in stem cells. This leads to a hypothesis that many stem cell related genes expressed in particular tissues arose by duplication and specialization of stem cell related genes originally expressed in other tissues. Superfamilies with large rates of duplication in the vertebrate lineage may have functions related to the development of an increasingly complex organism, including the generation and control of tissue-specific stem cell pools. All results and data presented here are available and can be queried through a web server [21].

## Results

We applied our method to a set of DNA microarray data from 83 samples from mouse stem cells and derivatives. Samples included embryonic, hematopoietic, mammosphere, retinal, neurosphere, adipose, and muscle cells (Table 1). All data were obtained using the Affymetrix MOE430 platform (Affymetrix Inc.) and subjected to quality controls as previously described [7].

**Table 1:** Set of mouse samples selected for our analysis from StemBase

Class	Samples	Replicates	SampleIDs
Adipose derived stem cells	1	3	S199
Dermis derived stem cells	1	3	S200
Embryonal carcinoma	4	12	S129, S130, S131, S132
Embryonic	8	23	S219, S220, S164, S165, S166, S167, S168, S169
Embryonic fibroblasts	2	6	S180, S286
Embryonic stem cell differentiation	35	105	S153, S154, S155, S156, S157, S158, S159, S181, S174, S241, S175, S206, S207, S208, S209, S210, S211, S212, S213, S215, S216, S217, S242, S243, S244, S251, S252, S245, S250, S246, S247, S248, S249, S127, S128
Hematopoietic	12	33	S233, S234, S235, S236, S237, S147, S291, S292, S293, S294, S295, S296
Mammary	5	15	S255, S256, S311, S312, S313
Muscle derived stem cells	3	7	S274, S184, S197
Neural	3	10	S271, S272, S198
Osteoblast differentiation	7	21	S185, S186, S188, S190, S192, S194, S196
Retinal derived stem cells	2	3	S232, S240

Various stem cells (SC) are represented in the data set. All original data including sample and experiment descriptions are accessible from the StemBase homepage [64].

**Figure 1:**
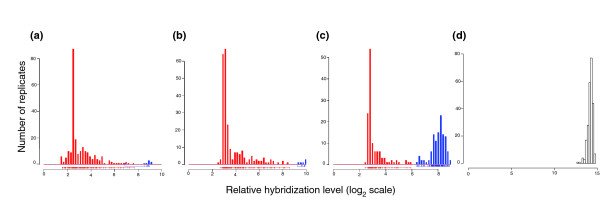
Distributions of hybridization values for probe sets. Each histogram depicts the number of replicates (from a total of 241) with a given hybridization value for a given probe set. For illustrative purposes, we display the distribution of hybridization values of three probe sets selected using the gap method as markers corresponding to **(a) **a known neural stem cell marker (Nestin; probe set 1449022_at), **(b) **a novel stem cell marker encoding a protein of known function (phospholipase Pla2g7; probe set 1430700_a_at) that we observed to be upregulated in bone marrow mast cell precursors and in undifferentiated mammospheres, and **(c) **a novel stem cell marker corresponding to an uncharacterized transcript upregulated in undifferentiated V6.5 and J1 murine embryonic stem cells (2410146L05Rik; probe set 1460471_at). Details on these three cases can be obtained through our online webserver [21] by viewing group numbers 73, 497, and 265, respectively. **(d) **For comparison, we show the distribution for a housekeeping gene (Eef1a1; probe set 1424635_at), a ribosomal translation elongation factor protein, which is always expressed and was not classified as a marker in our analysis. For robustness, the segregation of the samples (indicated by red and blue bars in the three marker distributions for the down and upregulated groups, respectively) is derived by analysis of the global set of patterns (see Materials and methods) and might not correspond perfectly to the distribution observed here. See, for example, a single replicate in the distribution of Nestin, which is left of the gap in the distribution but was (correctly) associated to the upregulated (blue) group.

At a U-score cutoff of 0.9 (see Materials and methods, below), our method identified 893 different two-state classifications with which to segregate the 83 sample dataset. Figure 1 shows three exemplar distributions of hybridization values for three probe sets that clearly segregate the dataset and are desirable choices for markers. Interpretation of the segregation pattern becomes obvious from the distribution itself as the group of samples with low gene expression values is separated from the group of samples with high expression values by a gap. Simplicity of interrogation allowed us to create a web tool (accessible on the internet [21]) to query these classifications so that users can determine whether a given gene is a marker, and find which samples it represents. Also, the tool allows us to find the markers separating two sets of samples of choice.

### Properties of the set of potential markers

**Figure 2:**
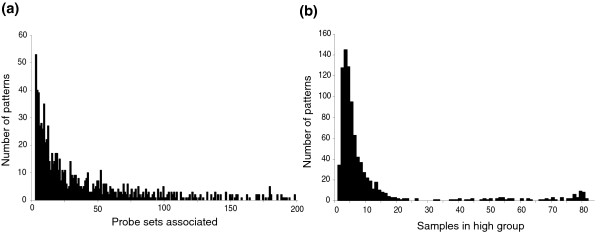
Properties of the set of 893 patterns. **(a) **Number of patterns with a given number of probe sets associated with a score of 90%. **(b) **Number of patterns with a given number of samples segregated in the high expression group.

Classifications contained varying numbers of probe sets. Figure 2a shows that patterns with small numbers of markers are more numerous. The 893 classifications also separated different numbers of samples into groups. Most patterns assigned small numbers of samples to 'upregulated' groups (for example, the gene was highly expressed in three samples versus 80). Over 80% of the patterns separate ten or fewer samples from the remainder of the database as a highly expressed group (Figure 2b). The complete set of putative markers associated with the 893 patterns includes 10,401 probe sets, or approximately 25% of the probes on the microarray, which is intuitively quite a large fraction.

We expected that patterns defined by smaller numbers of marker genes would be more likely to include genes important for stem cell related functions. To investigate this, we examined the distribution of known stem cell markers in this set and whether the method was able to select them preferentially within small clusters.

### Known stem cell markers in dataset

We investigated whether known stem cell markers were identified in our dataset in order to understand the properties and usefulness of the selected patterns within the context of stem cell research.

By examining the literature, we selected 88 marker genes that represented the variety of stem cell types in our dataset (Table 2). We identified the corresponding entries in the Entrez Gene database for 72 of the 88 marker genes. In seven of the remaining cases we were unable to identify definitively the correct gene because of ambiguity of the provided gene identifier (for example, 'laminin' could indicate one of several possible genes), and in nine cases the identifier could not be related to any entry in the database (for example, 'neuralstemmin'). Of these 72 Entrez Gene IDs, 71 had at least one associated probe set on the Affymetrix MOE430A/B chip set. Our set of 10,401 'potential marker' probe sets, which contains patterns with a maximum of 200 associated probe sets (described under Filtering marker lists by size and number of classified samples, below), contained probe sets for 49 of these 71 marker genes (69%).

**Table 2:** Stem cell markers

Ref.	Cellular type	Gene name	Entrez Gene	Probe set polled	Pattern	Score	D
[74]	Differentiated retinal	*309L*	*?*	-	-	-	-
		*Rho1D4 (rhodopsin)*	*?*	-	-	-	-
		*D2P4 (rhodopsin)*	*?*	-	-	-	-
		*CHX10*	*Chx10*	1419628_at.A	Not found	Not found	
		*PKC*	^a^	-	-	-	-
		*ROM-1*	*Rom1*	1448996_at.A	879	0.942	
	Photoreceptor specific homeobox	*Crx*	*Crx*	1418705_at.A	Not found	Not found	
	Muller glia	*10E4*	*?*	-	-	-	-
[75]	Human central nervous system	*CD24-/lo*	*CD24a*	1416034_at.A	573	0.987	2
		*CD34-*	*CD34*	1416072_at.A	72	0.990	2
		*CD45-*	*Ptprc*	1422124_a_at.A	427	0.987	
	Human neuronal lineage	*N-CAM*	*Ncam1*	1426864_a_at.A	318	1.000	
	Neural	*CD133*	*Prom1*	1419700_a_at.A	573	0.930	2
	Hematopoietic	*CD133*	*Prom1*	1419700_a_at.A	573	0.930	2
[76]	Proliferating neural	*Ki-67*	*Mki67*	1426817_at.A	8	0.991	
[77]	Trophoblast	*Cdx2*	*Cdx2*	1422074_at.A	Not found	Not found	
	Ectoderm	*Fgf5*	*Fgf5*	1438883_at.B	442	1.000	
	Neuroectoderm	*Isl1*	*Isl1*	1422720_at.A	83	0.931	
	Pluripotent stem cell	*Nanog*	*Nanog*	1429388_at.A	390	0.947	
		*Oct3/4*	*Pou5f1*	1417945_at.A	151	1.000	
		*Rex1*	*Zfp42*	1418362_at.A	547	0.964	
	Mesoderm	*Brachyury*	*T*	1419304_at.A	232	0.938	
[78]	Neural stem cell	*Hu*	*?*	-	-	-	-
		*Neuralstemmin*	*?*	-	-	-	-
		*ABCG2*	*Abcg2*	1422906_at.A	Not found	Not found	3
		*LeX/SSEA-1*	*Fut4*	1455843_at.B	771	0.924	
		*Musashi (Msi1)*	*Msi1*	1421409_at.A	Not found	Not found	2
		*Sox-1*	*Sox1*	1438729_at.B	542	0.958	
		*Sox-2*	*Sox2*	1416967_at.A	71	0.986	
[79]	Muscle specific	*MCK (muscle creatine kinase)*	*Ckm*	1417614_at.A	104	1.000	
		*MHC (myosin heavy chain)*	*Myh4*	1427026_at.A	Not found	Not found	
	Myofiber sarcolemma	*Dystrophin*	*Dmd*	1417307_at.A	835	0.958	
	Basal lamina	*Laminin*	^a^	-	-	-	-
	Myogenic lineage	*Myf5*	*Myf5*	1420757_at.A	Not found	Not found	
		*MyoD*	*Myod1*	1418420_at.A	32	1.000	
	Late myogenic lineage	*MRF4*	*Myf6*	1419150_at.A	Not found	Not found	
		*Myogenin*	*Myog*	1419391_at.A	104	1.000	
	Satellite cells	*Pax7*	*Pax7*	1452510_at.A	Not found	Not found	
[80]	Neural restricted precursors	*MAP2*	*Mtap2*	1434194_at.B	108	1.000	2
		*Beta3 tubulin*	*Tubb3*	1415978_at.A	Not found	Not found	
[81]	SKP	*Fibronectin*	*Fn1*	1426642_at.A	681	0.984	
		*GAP43*	*Gap43*	1423537_at.A	363	0.959	
		*MAP2*	*Mtap2*	1434194_At.B	108	1.000	2
		*Nestin*	*Nes*	1449022_at.A	73	0.980	2
		*p75NTR*	*Ngfr*	1421241_at.A	Not found	Not found	
		*Vimentin*	*Vim*	1450641_at.A	770	0.977	
[82]	Hematopoietic stem cell	*BCRP1*	*Abcg2*	1422906_at.A	Not found	Not found	
	Epidermal side population	*BCRP1*	*Abcg2*	1422906_at.A	Not found	Not found	3
		*alpha6-integrin*	*Itga6*	1422444_at.A	Not found	Not found	2
		*beta1-integrin*	*Itgb1*	1426918_at.A	13	0.967	2
		*keratin 14*	*Krt1-14*	1460347_at.A	386	0.983	
		*Sca-1*	*Ly6a*	1417185_at.A	637	0.929	2
		*CD34-*	*CD34*	1416072_at.A	72	0.990	2
		*E-cadherin*	*Cdh1*	1448261_at.A	48	0.993	
		*Keratin 19*	*Krt1-19*	1417156_at.A	268	0.989	2
		*CD71-*	*Tfrc*	1452661_at.A	845	0.958	
[83]	Muller glia; retinal	*Gln synthetase*	*Glul*	1426235_a_at.A	573	0.901	
	Retinal	*syntaxin*	^a^	-	-	-	-
		*Pax6*	*Pax6*	1419271_at.A	374	0.960	
		*rhodopsin*	*Rho*	1425171_at.A	Not found		
[84]	Neural	*(NeuN) Neuron specific protein*	*?*	-	-	-	-
		*Neuron-specific enolase*	*Eno2*	1418829_a_at.A	Not found	Not found	-
	Osteoblasts	*Alkaline phosphatase*	*Akp2*	1423611_at.A	Not found	Not found	-
		*BMP2*	*Bmp2*	1423635_at.A	Not found	Not found	-
		*BMP4*	*Bmp4*	1422912_at.A	Not found	Not found	-
		*BMP Receptor 1*	*Bmpr1a*	1425492_at.A	581	0.939	
			*Bmpr1b*	1437312_at.B	446	0.917	
		*BMP Receptor 2*	*Bmpr2*	1434310_at.B	602	0.954	
		*PTH receptor*	*Pthr2*	1452129_at.A	Not found		
		*Type 1 collagen*	^a^	-	-	-	-
		*bone sialoprotein*	*Ibsp*	1417484_at.A	Not found		
		*PTH receptor*	*Pthr1*	1417092_at.A	368	0.964	
		*RunX-1*	*Runx1*	1422865_at.A	295	0.982	
		*osteonectin*	*Sparc*	1448392_at.A	581	1.000	
		*osteopontin*	*spp1*	1449254_at.A	737	0.996	
	General stem cell factor receptor	*CD117*	*Kit*	1459588_at.B	243	0.912	
	Muscle	*merosin*	*Lama2*	1426285_at.A	799	0.986	
	Cartilage related extracellular matrix	*aggrecan*	*Agc1*	1449827_at.A	Not found	Not found	-
		*collagen II*	^a^	-	-	-	-
		*collagen IV*	^a^	-	-	-	-
		*PRELP*	*Prelp*	1416322_at.A	231	0.958	
	Adipose derived stem cell	*CD49d+*	*Itga4*	1421194_at.A	Not found	Not found	-
		*CD106-*	*?*	-	-	-	-
[85]	Mammary stem cell	*Bmi-1*	*Bmi1*	Not on array	-	-	-
		*p21*	*Cdkn1a*	1424638_at.A	400	0.967	
		*CD49f*	*Itga6*	1422444_at.A	Not found	Not found	2
		*Cytokeratin 19*	*Krt1-19*	1417156_at.A	268	0.989	2
		*Sca-1*	*Ly6a*	1417185_at.A	637	0.930	2
		*Musashi (Msi1)*	*Msi1*	1421409_at.A	Not found	Not found	2
		*Cytokeratin 5/6*	*?*	-	-	-	-
[86]	Neural enrichment	*CD24*	*CD24a*	1416034_at.A	573	0.990	2
	Skin	*CD29*	*Itgb1*	1426918_at.A	13	0.970	2
[87]	Neural	*GFAP*	*Gfap*	1426508_at.A	249	0.924	
		*Neurofilament*	*Nefl*	1426255_at.A	151	0.935	
		*Nestin*	*Nes*	1449022_at.A	73	0.980	2
	Photoreceptors	*Recoverin*	*Rcvrn*	1450215_at.A	Not found	Not found	-
	Epithelial lineage	*Cytokeratin (904-clone 34betaB4?)*	^a^	-	-	-	-
		*CK18*	*Krt1-18*	1448169_at.A	83	0.917	
		*AE1*	*Slc4a1*	1416464_at.A	41	1.000	
[88]	Epithelial lineage	*AE3*	*Slc4a3*	1418485_at.A	391	0.931	

Note that some markers may be included in multiple rows of the table (as many as indicated in the right-most column), because a number of genes have been identified as markers of more than one cell type (for example, *Abcg2 *for hematopoietic and neural cells [78,82], or *Nestin *for neural and skin-derived precursors [SKPs] [81,87]). Some of the markers could not be assigned to an Entrez gene either because the marker name was ambiguous (indicated by '^a^') or was absent from the database of gene names (indicated by '?'). Patterns can be examined online via the web server [21]. All patterns for which a polled probe set is a marker can also be examined at the web server. Note that the chip containing the probe set is indicated by '.A' or '.B' appended to the identifier, because some probe sets are included in both the MOE430A and the MOE430B chips.

**Figure 3:**
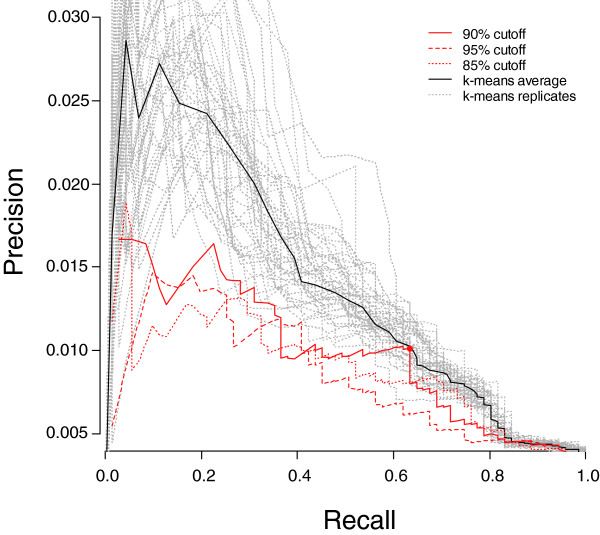
Precision/Recall curves for genes selected by the gap method and k-means. Precision/recall curves in red are associated with the gap method, and the point marked with a circle denotes the precision/recall associated with patterns associated with 63 probe sets or less. Gray curves show precision/recall for all replicates of k-means clustering, and the average expected precision/recall curve is shown in black. Recall values are based on the 71 stem cell markers defined in Table 2, whereas precision is the fraction of marker genes identified in the total number of predicted marker genes.

As previously observed, we obtained numerous patterns that segregate small subsets of samples and that are defined by small numbers of genes. To test our hypothesis that these would tend to contain relevant genes (in this case, genes useful for characterizing stem cells), we examined the recall and precision of our method for the 71 known markers as the maximum marker list length was reduced from 200 to 3 (Figure 3). Limiting the list to patterns defined by 63 or fewer markers reduced the total number of probe sets assigned marker roles from 10,401 to 5,848 (44% reduction) while losing only four known stem cell markers, representing a recall rate of 63% (45 out 71; point marked with a circle in Figure 3). This supports our theory that marker genes are more often contained in small clusters.

The 705 patterns defined by 63 or fewer markers segregated a mean of nine samples (median 5) in the upregulated group and were associated with a mean of 21 markers (median 15). This set associates 5,848 probe sets (4,449 genes) with at least one pattern, accounting for approximately 13% of the probe sets on the MOE430 microarray platform. We propose that many of those can be developed into useful markers. We define this as 'selected marker' set.

We compared the performance of our method with a popular method for analysis of gene expression data, namely k-means, which is a standard clustering algorithm (see Materials and methods, below). Both methods performed similarly in producing groups of genes that are expected to be enriched for stem cell markers (Figure 3). However, our method differs from a clustering algorithm in that we identify markers that segregate sets of samples whereas clustering algorithms group markers with similar expression patterns. Accordingly, the groups of associated markers produced by the gap method were somewhat different from the clusters obtained using k-means (mean overlap of 69.8%).

### Overview of the selected marker set

**Figure 4:**
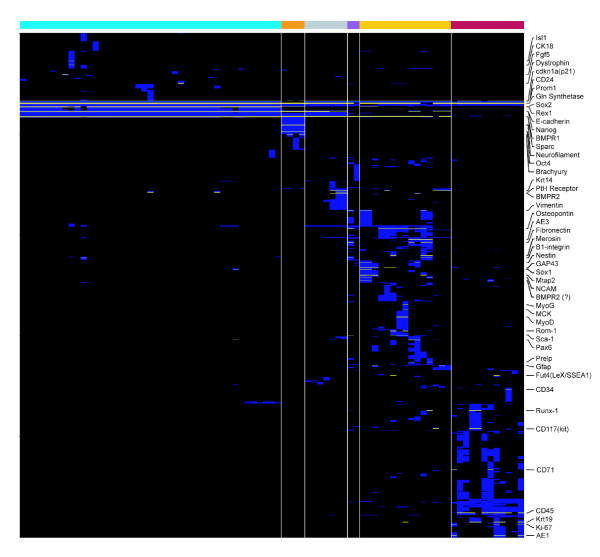
Heatmap indicating the distribution of patterns for markers. The horizontal axis shows 241 mouse samples used in this study. The vertical axis shows patterns for 1,301 markers either predicted to have high reliability (scoring 100%, *n *= 1,252; blue) or probe sets belonging to genes ascribed marker roles based on evidence in the literature (*n *= 49; yellow). Rows were clustered and diagonalized. Vertical separators were used to distinguish major sample cell types. Sample identities are grouped as follows: embryonic, blue; P19 embryonal carcinoma, orange; fibroblasts, purple; spheres, yellow; hematopoietic, red. Gene names are indicated only for the 49 stem cell markers.

To illustrate the variety of patterns identified, Figure 4 shows the expression patterns of the 49 probe sets that represent previously known stem cell marker genes identified by the algorithm (yellow), together with another 1,252 genes that were assigned a perfect score by the algorithm (blue). All major divisions in the dataset appear clearly defined, with samples related to one of hematopoietic, sphere [22], or embryonic sample types forming major groups. For example, hematopoietic samples define many patterns with probe sets identified uniquely within them.

### Gene Ontology statistics of the selected marker set

Since the selection of markers was done without reference to the identity of the samples, we would expect to find not just stem cell markers but also general markers of cell and tissue identity (for example, distinguishing differentiated blood cells from epithelial cells). To investigate in general terms the function of the genes that were selected in the marker set, we collected the 5,848 probe sets defining the 705 selected patterns (with 3 to 63 associated probe sets). These corresponded to 4,449 Entrez Gene IDs, for which we determined the over-representation of Gene Ontology (GO) annotations. Using the 'potential marker' set of 7,478 genes (which defined groups with up to 200 probe sets) as a reference, we found several significantly enriched functional categories (Table 3).

**Table 3:** Gene Ontology terms of marker sets

All	Selected set		High in differentiated		GO	GOID
N1	N2	P2	N3	P3		
7,478	4,449		222		Total	
976	727	2.85 × 10^-22^	81	1.60 × 10^-16^	Extracellular region	GO:0005576
169	131	0.001824	17	0.009344	Extracellular matrix	GO:0031012
72	57	>1	12	0.00106	Enzyme inhibitor activity	GO:0004857
2,061	1,390	8.07 × 10^-15^	73	>1	Membrane	GO:0016020
865	618	2.44 × 10^-11^	25	>1	Signal transducer activity	GO:0004871
587	421	4.00 × 10^-7^	15	>1	Receptor activity	GO:0004872
925	637	8.62 × 10^-7^	43	>1	Multicellular organismal development	GO:0007275
312	235	6.34 × 10^-6^	16	>1	Defense response	GO:0006952
911	614	0.000403	32	>1	Cell communication	GO:0007154
270	201	0.000485	17	>1	Cell adhesion	GO:0007155
436	310	0.000596	18	>1	Organ development	GO:0048513

Column labels are as follows: N1 is the number of genes for a Gene Ontology (GO) category in the unselected marker set; N2 and P2 are the number of genes and P value for the smaller marker set; N3 and P3 are the number of genes and P value for the markers highly expressed in differentiated stem cells; GO is the description of the GO term; and GOID is the GO identifier. P values are computed using the 'All' set of markers as background. GO terms displayed if they represent more than ten genes have at least one associated P value below 0.01 and do not overlap more than 80% with another displayed term.

Significantly enriched functions (*P *< 0.001) include those that allow cells to interact and respond to environmental cues ('defense response', 'cell communication', 'signal transducer activity', and 'receptor activity'), to interact with the immediate neighborhood of the cell ('extracellular matrix region', 'extracellular matrix', 'membrane', and 'cell adhesion'), and functions related to development ('multicellular organismal development' and 'organ development'). Finding these development-related functions is reasonable given that our set of samples is focused on stem cells. The abundance of functions related to the interaction of cells with their environment, on the other hand, may generally reflect that cell identity is largely defined at their surfaces; we would expect to see these functions in the analyses of other collections of gene expression data sampling multiple tissues.

### Examination of markers of stem cell differentiation

One distinctive feature of the gap method is that genes are selected based on their ability to define binary groupings of samples. This is meaningful and often desired from the point of view of an experimental researcher. Understanding the significance of the marker becomes simpler as the pattern itself gives a classification of the sample set.

Likewise, the identified sample partitions allow simple, direct, and intuitive ways to manipulate the gene expression data. We illustrate this here by applying a selection procedure to the set of patterns obtained above to focus on markers that are active in any of several lineages of stem cell differentiation included in our dataset.

Generally, we are interested in the properties of probe sets that separate undifferentiated and differentiated sets of stem cells. We chose six pairs of stem cell samples and their differentiated derivatives from our dataset (two embryonic stem cell lines, hematopoietic stem cells [HSCs], osteoblasts, mammospheres, and mast cell progenitors; Additional data file 3) and selected probe sets that segregated at least one sample pair with high confidence (99% association score); specifically, the probe set exhibited high expression in the undifferentiated sample and low expression in the differentiated sample, or *vice versa*. This selection identified 488 probe sets (called the 'stem cell related' set) corresponding to 426 genes, of which 206 exhibited high expression in the undifferentiated sample and 222 in the differentiated counterpart. Two genes showed high expression in the undifferentiated sample for some cell types, and in the differentiated sample in others: *Ugt1a2 *detected by probe set 1426260_a_at, and a gene encoding a hypothetical coiled-coil domain-containing protein detected by probe set 1444761_at. This set of 426 genes included five out of the 71 known stem cell markers used for benchmarking (*Krt1-14*, *Mtap2*, *Ncam1*, *Spp1*, and *Vim*).

Examination of the GO terms of the set resulted in a very short list of significant functions (*P *< 0.01). Separate analyses of genes upregulated in undifferentiated and differentiated states failed to identify GO terms over-represented in the genes that were highly expressed in stem cells. The only relevant terms for the genes expressed in the differentiated genes (Table 3) were related to the extracellular environment ('extracellular region' and 'extracellular matrix'), but with less statistical significance than those for the selected list of 4,449 cell markers. It is known that stem cells often rely on the maintenance of a stable microenvironment (the niche) for physical support and extrinsic cues (for a review, see Li and Xie [23]). The GO term 'enzyme inhibitor activity', not relevant for the larger marker set, appeared as relevant (*P *= 0.001) for a set of 12 genes, five of which belonged to the family of serpins (SERine Protease INhibitors; serpins a1b, a3n, b9, g1, and h1).

Serpins are a large class of proteins that are found in all multicellular eukaryotes and predominantly function as serine protease inhibitors, but they can also function as caspase and cysteine protease inhibitors and, in rare cases, as hormone transporters, chaperones, or tumor suppressors. In contrast to eukaryotes, prokaryotic serpins are rare and most serpin-containing prokaryotes have only a single serpin gene [24]. In agreement with our findings, variation in serpin gene expression was recently observed during differentiation in the myeloid lineage [25,26]. Here we give some insight into the functions of the six serpins we identified in our study.

Serpinb9 is an estrogen-inducible caspase inhibitor that can inhibit granzyme B-mediated apoptosis, a key mechanism by which cytolytic lymphocytes are able to destroy target cells. It is expressed at high levels in testis and placenta, and may contribute to the ability of immune-privileged cells to evade destruction [27]. Recently, it was shown that serpinb9 plays a role in allowing embryonic stem cells (ESCs) to evade a similar fate [28]. Serpins a3n and a3g have been shown to be strongly influenced by LIM-homeobox 2 expression in a hematopoietic system [29].

Serpina3g was previously reported to be highly enriched in HSCs, in concordance with our observations [30]. Similar in function to serpinb9, serpina3n has been implicated in providing protection from granzyme B mediated cell death in a study of Sertoli cell secreted factors [31]. Interestingly, Serpinh1 is one of the exceptions of the serpin family; it is a chaperone molecule that plays a role in the maturation of pro-collagen [32]. With relevance to stem cell biology, Serpinh1 knockout ESCs produce embryoid bodies with aberrant morphology [33]. In summary, the abundance of serpins as markers might reflect generalized mechanisms of immune system avoidance that are activated in cells undergoing differentiation.

This complete set of cell markers offers a good basis from which to study principles of stem cell gene function. For example, of the 426 genes selected as markers, a small number of genes (17) were involved in several of the six lineages selected (at least one of them being non-ESC). Of those, nine were highly expressed in the stem cells, six were highly expressed in the differentiated partners, and only two were expressed in both (Additional data file 3). This indicates not only that a single gene can be involved in multiple stem cell differentiation lineages but also that if it does then it will most often act in a similar way across those lineages.

**Figure 5:**
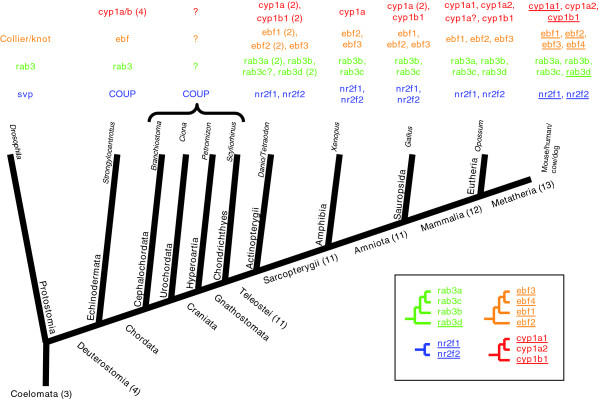
Phylogenetic distribution of stem cell markers and their close paralogs in four protein families. Major taxa along the Coelomata lineage are depicted in bold black text with deduced numbers of genes from these families in parentheses. Phylogenetic relations between species were taken from the National Center for Biotechnology Information's Taxonomy Browser, except for the relation between Cephalocordata and Urochordata, which was taken from Vienne and Pontarotti [72]. Species names are given in italic black text. Colored text indicates paralogs for four families: red for *cyp1*, orange for *ebf*, blue for *nr2f1*, and green for *rab3*. Numbers in parentheses indicate multiple copies of gene (for example, in *Actinopterygii *genes). Many genes are duplicated in *Actinopterygii *because of a whole-genome duplication event postulated to have occurred along the ray-finned fish lineage [73]. Most expansion of these four families occurs after divergence of Deuterostomia and before divergence of Teleostei. Underlined genes for mouse indicate genes identified in the selected marker set. The database identifiers of the sequences are given in Additional data file 4. The inset on the lower right corner shows schematic phylogenetic trees for the murine members of the four families. All branches shown had bootstrap values above 0.5. Outlier sequences used for each family (not displayed): *D. melanogaster rab3 *for rab3, *S. purpuratus cytp1 *for cytp1, *D. melanogaster svp *for nrf2f1, and *D. melanogaster knot *for ebf.

This set allowed us also to study stem cell evolution. We were interested in determining whether there would be a relation between sequence similarity and involvement in stem cell function and involvement in one or many lineages. Large gene families with frequent duplication and reuse are valuable in these investigations because the members will have varying degrees of sequence similarity. Our set of stem cell markers provides a starting point to search for these families. To identify superfamilies within our set of stem cell markers, we performed an exhaustive pairwise sequence comparison of the protein sequences of the 426 stem cell markers (see Materials and methods, below, and Additional data file 3). Manual examination of the results to select full length similarity identified four superfamilies containing three or more members: serpins (cluster #18 in Additional data file 3), the nuclear receptor family (cluster #4), the cytochrome P450 family (cluster #17), and the Rab family GTPases (cluster #10). As serpins are described above, we opted to investigate further members from the additional families within the context of gene evolution in relation to stem cell function and expression pattern (Figure 5).

#### Nuclear receptors: Nr2f2

The proteins of the family of nuclear steroid and hormone receptors are dimerizing transcription factors characterized by a DNA binding domain and a carboxyl-terminal hormone binding domain; they are implicated in cell proliferation, differentiation, and apoptosis [34]. Three members of this family were identified in the set of 426 stem cell markers (*Nr2f2*, *Essrb*, and *Rora*). We examined *Nr2f2 *in greater detail.

**Figure 6:**
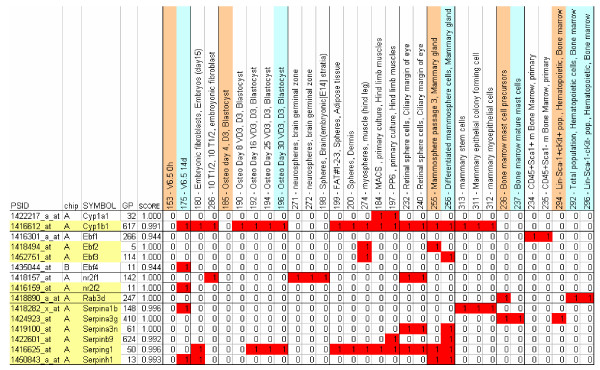
Sample segregation for selected markers. Samples segregated in the high expression group for probe set markers from the nuclear receptor (*nr2f1 *and *nr2f2*), cytochrome P450 family (*cytp1a1 *and *cytp1b1*), serpin family (*Serpins a1b*, *a3g*, *a3n*, *b9*, *g1*, and *h1*), and Rab GTPase (*rab3d*) superfamilies. The *nr2f1*/*nr2f2 *and *cytp1a1*/*cytp1b1 *gene pairs are highly similar in sequence but their expression patterns are notably different. Undifferentiated and differentiated sample pairs are shaded in pink and blue, respectively.

Nuclear receptor subfamily 2, group F (Nr2f)2/COUP-TF2 represses Notch signaling activity in determination of vein identity [35], but it is also expressed in multiple tissues and organs of the embryo and is required for early outgrowth of limb buds [36]. In our set of samples, probe set 1416159_at, which detects this gene's transcript, segregates the V6.5 differentiated murine embryonic stem cell (mESC) sample from the rest (Figure 6). The 80% identical Nr2f1/COUP-TF1 (detected by probe set 1418157_at) segregates the samples of retinal spheres, neurospheres, and 10 T1/2 embryonic fibroblasts, but not any of V6.5 or other mESC samples, differentiated or not. By contrast, the probe sets for another close paralog Nr2f6/COUP-TF3 (1460647_a_at and 1460648_at) were not identified as markers.

The genes *nr2f1 *and *nr2f2 *share a common ancestral gene that is represented in the fly. The *Drosophila *homolog of *nr2f1 *and *nr2f2*, *svp *(seven up), regulates stem cell identity of neuroblasts in order to control the identity of differentiated progeny cells [37]. In this case, there is a conservation of involvement in stem cell function from the divergence between Protostomia and Deuterostomia. Phylogenetic analysis (Figure 5) indicates that the gene is conserved as a single copy, possibly until divergence of Gnathostomata. All Teleostei appear to have the duplicated version of the gene. However, the patterns of gene expression in stem cells are very different (Figure 6), indicating the specialization of the duplicated copies of the gene.

#### Cytochrome P450 family: Cyp1b1

Cytochrome P450 proteins (CYPs) are a family of enzymes that are present in bacteria and Eukarya that participate in the metabolism of exogenous or endogenous chemicals [38,39]. Four members of this gene family were identified in the set of 426 stem cell markers (*Cyp1b1*, *Cyp24a1*, *Cyp4f18*, and *Cyp7b1*). All four were highly expressed in various differentiated cells and expressed at a low level in their undifferentiated counterparts. We conducted a detailed analysis of *Cyp1b1*.

Phylogenetic analysis of Cyp1b1 (Figure 5) suggests the existence of two very close paralogs: Cyp1a1 and Cyp1a2. No equivalent sequence in the *Drosophila *genome (or in any other Protostomia) was identified, but the Echinodermata *Strongylocentrotus purpuratus *(purple sea urchin) appears to have an ancestral *Cyp1a/b *gene (with four copies, possibly duplicated after its divergence from Chordates). The ancestral gene appears to have duplicated before divergence of Actinopterygii from Sarcopterygii into the 1a and 1b forms, and the subsequent duplication of the 1a form appears to be absent in Sauropsida (for example, chicken) but present in all mammals (for example, opossum). In birds *Cyp1a *seems to have undergone a separate duplication after divergence from mammals.

In our set, *Cyp1b1 *segregates differentiated osteoblast cells and differentiated mammospheres from the rest of the dataset. Cyp1b1 metabolically activates estradiol (to produce 4-hydroxy estrogens), which are able to induce estrogen receptors, and mutation of Cyp1b1 may stimulate estrogen-mediated carcinogenesis [40]. It has also been suggested that Cyp1b1 is involved in axis control during embryonic development [41].

By examination of the larger set of markers, we can see that Cyp1a1 is a muscle stem cell marker, but its paralog, Cyp1a2, does not behave as a marker in our dataset. This is supported by the observation that *Cyp1a2*^-/- ^null mutant mice develop normally with just some deficiencies in drug metabolism [42]. To the contrary, *Cyp1a1 *is potentially involved in many cancers and might also have a function in murine embryonic development [43]. CYP1A2 is one of the major CYP1 enzymes that catalyze 2-hydroxylation of estrogen [44], but the substrate of CYP1A1 is not yet known.

*Cyp1a1 *and *Cyp1a2 *are transcribed from the same bidirectional promoter region [45]. Their head-to-head arrangement is conserved in mammalian genomes, which suggests that the genomic organization of these genes is of functional significance. The fact that these two genes have different behavior as stem cell markers indicates that there are factors uncoupling their expression.

#### Rab family of GTPases: Rab3d

The Rab family of small GTPases are involved in intracellular cell signaling processes, including tethering and docking of vesicles to their target compartment, vesicle budding, and interaction of vesicles with cytoskeletal elements [46]. According to SMART (Simple Modular Architecture Research Tool; 4 April 2007), there are 66 mouse Rab proteins (defined as containing a Rab domain and no other annotated domain). We identified three members of this family in the set of 426 stem cell markers (*Rab3d*, *Rab31*, and *Rab38*). *RhoJ *was detected by sequence similarity but discarded after manual examination because it belongs to a different family. We performed a detailed analysis of *Rab3d*. In our set of markers *Rab3d *expression segregates mast cell precursors. None of its paralogs, *Rab3a*, *Rab3b*, and *Rab3c*, was identified as a marker by our methodology.

The ancestral *Drosophila *gene, *Rab3*, is expressed in the nervous system [47]. Echinodermata *Strongylocentrotus purpuratus *has only this ancestral gene (Figure 5), but all Teleostei have four copies of the gene, suggesting duplication after the divergence of Chordata and Echinodermata.

In agreement with *Rab3 *expression patterns in the fly, the four *Rab3 *paralogs are expressed in mouse brain, where they regulate vesicular release; genetic deletion of individual paralogs does not affect viability or fertility in mice, but knockout of all four genes results in early perinatal mortality [48]. However, these genes are expressed elsewhere. For example, *Rab3a *is detected in acrosomal membranes of mouse sperm [49]. *Rab3d *is expressed in the exocrine pancreas and the parotid gland, where it is involved in secretory granule maturation [50]. Finally, *Rab3b *and *Rab3d *are expressed in mast cells [51], which explains our observation that *Rab3d *segregates mast cell precursors.

#### Early B-cell factors: Ebf2 and Ebf3

In each of the four superfamilies analyzed above (serpins, nuclear receptors, cytochrome P450, and Rab GTPases), we note that most members exhibited the same gene expression behavior along differentiation (being highly expressed in either stem cells or in their differentiated counterparts). However, this is not the general case. If we consider all 49 clusters of protein sequences (Additional data file 3), about half (26 of 49 [53%]) have some family members that are highly expressed in stem cells and others that are highly expressed in the differentiated counterparts; the remaining 23 are highly expressed in stem cells (10) or are highly expressed in differentiated cells (13). An example of the former is given by the two early B-cell factors Ebf2 and Ebf3, which we study here in detail.

The four mouse members of the Ebf family of helix-loop-helix transcription factors have non-redundant adipogenic potential in multiple cellular models [52]. Interestingly, both *Ebf2 *and *Ebf3 *are detected in our set, but with opposite effects. *Ebf2 *is upregulated in mammospheres and *Ebf3 *in differentiated mammospheres. The other two mouse members of this family, namely *Ebf1 *and *Ebf4*, are detected in the selected marker set. *Ebf1 *(probe set 1416301_a_at) is identified as segregating several bone marrow samples. *Ebf4 *(probe set 1435044_at) is identified as segregating differentiated V6.5 mESCs [53]. This family constitutes an example of genes arising by duplication of an ancestral gene (represented in *Drosophila *by the Collier/knot transcription factor, which is involved in Hedgehog patterning [54] and control of hematopoiesis [55]), with multiple and varied stem cell related functions arising as the gene duplicates.

## Discussion

We have developed an unsupervised approach to identifying coordinately acting biomarkers using heterogeneous microarray data, which can be generalized to any set of gene expression data regardless of the platform on which they were generated. This method is a ground-up approach that first determines the extent of the information available in a set of array data through a discretization step to classify samples and initialize patterns. Genes are then associated with patterns based on the presence of a clear demarcation threshold in their expression values, which can reproduce the classification of samples. These genes can therefore act as markers for the samples segregated in the subset.

Microarray technology has followed a trend toward increased feature density and increased coverage, and is customarily used to address large exploratory questions. Our development of this method was motivated by the desire to present experimental groups with information that clearly shows which genes differentiate samples of interest within the larger context of many samples in the database (for instance, which genes are upregulated in my samples of interest?), and what other samples are similar to these samples of interest (in which other samples are these genes upregulated?).

By identifying probe sets that exhibit bimodal expression level patterns, we directly cater to researchers wishing to assess the significance of particular genes in conditions of interest through methods such as polymerase chain reaction or northern blotting. The interactive graphical presentation of expression values as distributions is exceptionally effective for assessing the ease with which a proposed marker gene might be validated, and the samples that it can be used to identify.

In the application we present, our approach focuses on probe sets. As genome annotations have changed, probe set annotations may be altered, changing the probe set to gene mapping or the annotated gene functions. This is understood to be an intrinsic problem in array design [56], and many probe sets on Affymetrix arrays are of unknown identity or are sparsely annotated. However, these obstacles do not render a probe set useless for the purposes of marker identification, because each probe set has a target sequence that is fixed at the time of array design. This sequence can be used to detect the molecular RNA species that is acting as a marker. Furthermore, the obvious inference when using cDNA microarrays is that the associated genes can function as markers at the protein level. Identification of a marker probe set implies the existence of a molecule (mRNA or protein) that can potentially be used as a molecular marker to discriminate between cell types. Even if probe set annotations are missing or inaccurate, follow-up experiments can be performed to validate or identify the molecules involved and determine whether development of an mRNA or protein molecular marker is feasible.

Our gap method stands apart from existing methods of microarray clustering. Standard clustering algorithms such as hierarchical or k-means clustering have two commonly raised impediments. The first is that both methods group genes based on global similarity of expression patterns. That is a restrictive test, which is not necessarily useful in the search for a marker. Second, and more importantly, both methods assign each gene and condition to a single cluster, which may not always be desirable. Proteins can have multiple functions in different contexts. In general, these clustering methods are best used when trying to identify co-regulation or co-expression of genes in a series of samples that are relatively homogeneous. In contrast, we are striving not to identify strict co-expression of many genes on a global level but, rather, to identify sets of genes with expression level thresholds that demarcate similar sets of samples in a heterogeneous microarray dataset. This selection procedure is more appropriate to the selection of markers.

Previously established methods also study gene expression values across sets of samples to identify biomarkers. Our method has some differences from those methods that we believe make it more useful for some applications. The methods from Pepe and coworkers [57], SAM (significance analysis of microarrays) [10], and PAM (prediction analysis for microarrays) [58] require the investigator to separate the samples into two classes; this might be appropriate for simple situations but it would be cumbersome for a dataset with multiple cell types and conditions, especially if a researcher is exploring novel ways to arrange the data. Our unsupervised classification method is more appropriate for such a case.

Like our method, the method presented by Beattie and Robinson [59] can produce patterns in an unsupervised manner. However, the binary patterns are obtained through an analog to digital transformation via a one-dimensional clustering step. We believe that our use of a threshold value to discover binary patterns is more intuitive for experimental biologists, and will simplify the development of following validation studies. As explained above, a clear demarcation of expression level between the two groups influences the decision for follow up more than the overall structure of the data in each state. Ideally, we would like to present candidates that also exhibit large fold changes with high statistical significance. The logic of using a threshold value that is directly related to the relative mRNA concentrations represents a margin of safety for the detection of differential expression, and is commonly illustrated by the use of a 'twofold rule' in the literature. Put simply, a biologist is more likely to follow up on a candidate marker that has clear separation of expression level, over other candidates that may have better statistical support but would pose a challenge for validation because of a tight range of expression.

Furthermore, the approach of Beattie and Robinson [59] assembles clusters by combining genes that generate identical binary patterns. Our observations indicate that the level of noise in gene expression data must be accounted for and some degree of fuzziness must be allowed in any analysis. Essentially, one stray sample should not ruin the association of two genes across a large number of samples (here, 83). Logically, the possibility that such stray hybridizations are encountered increases with increasing dataset size. One such example is illustrated in Figure 1a. We believe that cluster formation by a measure of discrimination that is more resilient to small degrees of overlap in the distributions (caused by experimental error) will yield more fruitful results and that adhering to matches between digital patterns for cluster formation may be unnecessarily strict. The original data can be always retrieved and a closer examination can be used to verify whether the classification suggested by each probe set is true. This allows genes to be detected as markers of similar patterns.

To illustrate the usefulness of our methodology in cases in which unsupervised patterns with resilience to noise are needed, we applied the method to a database of 83 samples from a variety of mouse stem cells and derivatives. Our results indicate that this method produced a list of candidate markers (selected marker set) that was fivefold enriched for a set of 71 known stem cell markers; we identified 45 of these while reducing the total number of probe sets under consideration from 45,137 to 5,848.

To verify the performance of our method, we clustered the dataset using k-means, a standard clustering algorithm (see Materials and methods, below), and found the precision and recall values to be similar. The overlap of results with the k-means clusters was appreciable (69.8%). However, our method cannot be considered as simply a duplication of the results of k-means, because our method groups genes by their ability to segregate the database and not by the similarity of their patterns of expression. Another important difference is that our method generates groupings of samples while identifying markers - something that k-means lacks. If one were to choose to develop marker genes from the results generated with k-means, a subsequent analysis of the expression profile in each k-means cluster would be required to identify the samples in which the cluster of genes might be more highly expressed. This can be done with a single set of genes but would require an additional automated method to process all k-means clusters if a meta-analysis were desired (such as our identification of genes related to differentiation in multiple cell types). Thus, the gap method presented here provides important additional data that is useful for a variety of subsequent explorations.

The selected marker set of genes was enriched in genes with particular features: products located on the exterior of the cell, and functions related to cell communication and differentiation (Table 3). This could be expected because our collection of data contains samples from diverse cells and tissues, and cell identity is mostly related to the cell surfaces and to the ways in which cells interact with their environment, including communication with other cells. In addition, because our dataset is enriched for differentiating tissues, a number of major functions related to development appeared in the list.

Although the selected set of markers is somewhat biased toward the objectives of analysis of stem cells, it also includes markers that, for example, distinguish one lineage from another (for instance, adult versus embryonic expressed genes). Our methodology facilitates focusing attention toward markers for samples that one is interested in. Here, we focused a more detailed analysis on markers segregating at least one of a short list of six differentiation lineages from the set of 83 samples. The use of these 83 samples as background increases the likelihood of identifying such specific markers.

A total of 426 genes were identified as stem cell related markers for the six differentiating lineages. Functional analysis revealed fewer statistically over-represented functions than in the selected set of markers. Analysis of the 222 genes segregating the differentiated samples (Table 3) revealed that the only over-represented GO annotations also significant for the selected set of markers were the association of genes to the extracellular region and the extracellular matrix. It is known that extracellular interactions are important between stem cells, their progeny, and their immediate environment in the maintenance of the stem cell state, control of stem cell populations, and associated progeny [23]. The one GO annotation over-represented only in this set was enzyme inhibition (for 12 genes). This annotation is represented in the list by five members of the serpin superfamily. A literature search suggests that these genes may play a role in suppression of immune system effects against differentiating cells. The absence of other functional enrichments suggests that there may be no specific gene function that endows cells with the property of 'stemness', in the same way that there appears to be no single stemness gene for all stem cells [60].

This set of 426 markers of stem cell differentiation allowed us to make some general observations regarding stem cell function and evolution. First, we observe that if a gene is a marker for differentiation in multiple lineages, then it will act the same way in most cases. Of the 17 genes identified as markers in multiple lineages, only two were found to be highly expressed in one differentiated population and in another undifferentiated population.

To examine whether patterns of expression in stem cells were retained for gene homologs, we studied gene families represented in the set of markers. Identification of 49 clusters of related protein sequences indicated that gene expression behavior was not conserved within those families because more than half of the clusters (26) included genes with conflicting gene expression segregation properties. However, we also wanted to study how expression patterns in stem cells were conserved with protein sequence identity. For this we looked for the largest families within the set that reflect a large number of gene duplication events and therefore offer multiple levels of sequence identity between their members. We selected four families with two to four true paralogs in the marker set: nuclear receptors, cytochrome P450s, Rab GTPases, and early B-cell factors. These superfamilies share general functional activities, but they have obtained additional functional or tissue specificities through mutation and selection following gene duplication. Phylogenetic analysis of one example from each family (Figure 5) led to the study of the evolution of a total of 13 genes. An ancestral gene existed for each of the four families before divergence of Deuterostomia, with three being present in the Protostome *D. melanogaster *(*knot*, *rab3*, and *svp*). The genes *svp *and *knot *are involved in stem cell differentiation. The two genes arising from duplication of *svp *and the four genes arising from duplications of *knot *are all detected as markers in our selected marker set. Similar observations are made for the gene *Cyp1a/b*; this gene underwent two duplications to produce a family of three genes, with two involved in differentiation and identified in our set of markers. We propose that the ancestral versions of this gene (for example, in *S. purpuratus*) are involved in differentiation. The *Rab3 *family illustrates a case in which duplication of an ancestral gene (which has synaptic functions in *D. melanogaster*) produces *Rab3d*, which plays a role in mast cell development, and *Rab3a*/*Rab3b*/*Rab3c*, which we do not identify as differentiation-related markers. The four murine genes conserve some neural function like that of the ancestral *Rab3*, but they appear to have obtained other functions and tissues in which they are expressed. Based on the results above we can hypothesize that the duplication of a gene involved in a development related function is likely to result in genes that are also involved in development.

Our second observation is that, in contrast, the range of expression is not necessarily conserved between close paralogs. *Ebf2 *and *Ebf3*, for example, are highly expressed in undifferentiated and differentiated mammospheres, respectively. Other cases can be observed in Figure 6. We did not identify redundant markers, that is, paralogous genes with the same segregation properties.

Our third observation is that clusters of genes with stem cell related functions appeared during a window of evolutionary time. Expansion of gene families associated with developmental functions is demonstrated by the number of paralogs of each family present in different organisms (Figure 5). Of 13 genes from the four families, three are present in *Drosophila *(*svp*, *Rab3*, and *knot*), four in *S. purpuratus *(*COUP*, an ancestral version of *Cyp1a/b*, *Rab3*, and an ancestral *Ebf*), and probably 11 in Teleostei. By the time of the Metatherian divergence, all 13 genes are present and there are no subsequent duplications. A substantial portion of gene family expansions was complete by the divergence of Teleostei from Chondrichthyes, which agrees with our previous phylogenetic analysis of genes involved in mESC differentiation [61]. The implication of this is that use of model organisms such as *Danio rerio *(zebrafish) and *Xenopus *in stem cell research may yield insights that can be translated into mammalian systems, provided that the appropriate paralogous genes are chosen for study. Our analysis also suggests that the completion of the genomes of members of the Urochordata, Cephalochordata, Hyperoartia, and Chondrichthyes taxa will provide great insight into the evolution of genes that are involved in the regulation of cellular and tissue complexity, in particular of those genes related to stem cell differentiation.

With this analysis we identified many stem cell specific markers in parallel, allowing us to establish some concepts regarding the evolution of stem cells. This demonstrates the value of the new tools described in this work. The study of genes from our marker lists will allow identification of mechanisms and cell populations that together contribute to stem cell function in a variety of different tissues.

## Conclusion

We have demonstrated a method for detection of markers from heterogeneous collections of samples of DNA microarray data of gene expression. We have applied this method to a highly heterogeneous set of stem cell gene expression data, with the objective being to detect markers relevant to stem cells, which a specific contextual question. The gap method detected markers through the unbiased generation of secondary data, which facilitated directed analysis of the results.

We believe that our method is more appropriate for the identification of targets for biomarker development than standard analytical techniques such as hierarchical clustering, when applied to DNA microarray data. The gap method is generally applicable to other large heterogeneous datasets in which one desires to find markers that act in a small proportion of the samples.

## Materials and methods

### Environment

Manipulation of data and statistical calculations were performed in the R language (version 2.3.1), available over the internet [62]. Packages implemented for biologic applications are available from the Bioconductor project [63], which runs in the R computing environment.

### Source of experimental data

Raw data in the form of Affymetrix CEL files for the appropriate samples were obtained from StemBase [64]. A subset of mouse samples based on the MOE430A/B DNA chip set was selected, encompassing samples from embryonic and adult stem cells and their derivatives. Expression values were generated from .CEL files using the GCRMA package [65] implemented in Bioconductor. Initial analyses allowed us to identify some samples whose values were in general outliers (because of nonstandard RNA preparation procedures). These were discarded and the expression values were re-computed with GCRMA on the reduced dataset. The data set used here contained 241 replicates (unique chips) derived from 83 different samples (of unique biological origin; Table 1). The expression signals were organized in a matrix with columns for samples and rows for probe sets, with the entries in the matrix representing the hybridization level.

### Generation of database partitions

To identify possible permutations of partitions for the expression matrix, we used a strategy to identify individual probe sets whose distribution of expression values appeared to have a break in continuity (a gap). This was motivated by the observation that known marker genes are expressed at two or more obviously distinct levels (see the example for Nestin in Figure 1a).

Briefly, for each probe set in the expression matrix, we ordered the expression values and calculated the differences between consecutive values. Large difference values suggest a demarcation in the expression values for a particular probe set, which may be the result of two underlying subpopulations of samples. If the difference exceeds a cut-off value, samples on either side of the gap are assigned to two groups (low/high expression) and encoded as binary vectors. The cut-off value used in this analysis was 50%, that is, a 1.5-fold difference, which on a log2 scale translates to approximately 0.6 units. This value was chosen to generate a number of partitions that did not produce an excessive rate of false positive clusters (see 'Filtering marker lists by size and number of classified samples', below). Note that we consider the possibility of finding more than one gap.

We used majority voting rules to correct each binary vector so that all replicates of any given sample were either 0 or 1. Ties were assigned a 0. For example, if two out of three replicates were assigned a 1, then the remaining replicate was also assigned a 1. This ensures that database queries corresponded to the underlying vectors used in the analysis. These corrected vectors were used to score probe sets in each cluster, and they were also used to generate visualizations on the web server associated with this work [21].

### Identification of markers for each partition

We identified groups of probe sets that can act as markers for each pattern of up/down regulation defined by the binary patterns as follows. For each binary vector (which represents one way of partitioning the database into two), we calculated Mann-Whitney U scores for each probe set on the microarray. The U statistics were calculated from the expression values in each group designated as highly expressed versus the expression values exceeding the 90^th ^percentile of the group with low expression. This modified U statistic is linearly correlated to the partial area under the curve described by Pepe and coworkers [57] at a false positive rate of 10% (data not shown). The advantage of using the U-statistic here lies in its decreased computational cost as compared with the calculation and integration of the area under the curve. The U scores were converted into a percentage of their maximum value for each group. Probe sets were ranked by these scores, and those exceeding 90% of the maximum U score were saved as candidate markers for each binary vector. The choice of this threshold was determined to produce satisfactory results according to the precision/recall curves computed below.

For illustrative purposes, this section is the most computationally intensive step and requires approximately 30 CPU hours to complete.

### Filtering marker lists by size and number of classified samples

Because our analysis examines thousands of probe sets, the analysis above is likely to identify many non-significant markers by chance. A simple way to eliminate those is to accept only sample partitions that are identified by multiple probe sets. To establish a lower threshold for the length of marker list to be accepted, we generated marker lists using a randomized version of the expression matrix. The expression values for each probe set were randomly reassigned to samples in order to destroy their biologic ordering while individually maintaining their range and distribution. We then generated marker lists for each binary vector as described above. Approximately 98% of patterns formed from the randomized matrix were associated with two or fewer probe sets, suggesting that such patterns are likely to arise by chance. Thus, in our analysis of the stem cell dataset we decided to report only patterns associated with three or more probe sets.

We also observed that the patterns with the largest numbers of probe sets identified major groups of tissue specific genes and therefore were not useful because they reproduced obvious sample partitions. For example, a cluster of 242 probe sets was identified that distinguished all blood related samples (hematopoietic and general bone marrow related) from others in the database. We therefore did not report clusters with more than 200 probe sets. This selection resulted in a list of 893 patterns involving a total of 10,401 probe sets for 7,478 genes. We define this as the 'potential marker set' (Additional data file 1).

### Calculation of precision/recall curves

In order to assess the method's performance in detecting 71 known stem cell marker genes (Table 2), we gradually decreased the upper limit of allowable cluster size in the dataset and calculated precision/recall values on the sets of clusters that passed the criteria at each step (Figure 3). This analysis was used to choose a 90% cut-off for generating clusters (used in 'Identification of markers for each partition', above) with the gap method, and to define a smaller set of markers (from the 705 patterns with 63 or fewer markers), the 'selected marker' set (Additional data file 2).

### Benchmarking of method with k-means clustering

In order to compare the gap method with an established method of grouping genes, we identified clusters of probe sets with k-means clustering according to their patterns of gene expression. We specified 1,000 clusters, which is similar to the number of clusters generated by the protocol described above, and selected the genes classified in those groups. The default algorithm for the 'kmeans' function in R was used. We computed the precision/recall values for k-means clustering by gradually decreasing the maximum allowable cluster size, as above. Because the k-means algorithm is not deterministic, to estimate the expected precision/recall we generated a mean precision/recall curve from 100 repetitions of k-means clustering. This curve is compared with curves generated using our clustering method in Figure 3.

We compared the overlap between the sets of genes selected using the gap method and k-means. For each of the k-means control runs, we selected the genes from the set of clusters that produced a recall of at least 45 known stem cell markers (this is the approximate recall level in Figure 3). The overlap between that k-means iteration and the gap method was computed as the fraction of genes selected by both methods divided by the total number of genes selected by either method. The mean overlap between the gap method and all k-means iterations was 69.8%.

### Enrichment of Gene Ontology annotations

We examined the functions of genes associated with each pattern by characterizing the enrichment of their GO annotations [66]. Because many genes are represented on the array by multiple probe sets, we mapped probes to their corresponding Entrez GeneIDs and calculated functional enrichment on a per gene basis. Affymetrix probe sets were mapped to Entrez GeneIDs using the 11 April 2006 release of NetAffx annotations [67]. Where probe sets had multiple GeneID mappings, the first one was selected because we observed that in the majority of such cases the first identifier tends to be the only one with a published symbol as opposed to one that was automatically generated. Links from GeneIDs to GO annotations were obtained from [68] on 9 May 2006 and traced up through the GO ontology to identify 'ancestral' terms. We calculated the cumulative hypergeometric *P *values for each GO annotation as per the method proposed by Tavazoie and coworkers [69]. Raw *P *values were then subjected to a Bonferroni correction to take into account the number of GO categories considered. These adjusted *P *values are reported in this analysis. The analysis of GO enrichment in the selected and high-in-differentiated sets of markers (Table 3) was done using the potential marker set as background.

### Representation of markers associated to samples

To generate an overview of the markers associated with different samples by the gap method represented in Figure 4, binary patterns were clustered with complete linkage hierarchical clustering based on their Euclidean distances. Major clusters were manually identified from the hierarchical tree and assembled into groups. Each row group was then associated with the column that contained the maximum value of the averaged binary vectors in the group. Row groups were then arranged by increasing column numbers.

### Analysis of protein families involved in stem cell differentiation

We chose six pairs of samples from our database representing undifferentiated and differentiated states of stem cells (Additional data file 3). We selected all probe sets segregating at least one of those pairs with a score exceeding 99%. These probe sets were mapped to RefSeq protein sequence IDs based on NetAffx annotations dated 12 July 2006. The 488 selected probes mapped to 420 RefSeq protein IDs, and these 420 protein sequences were obtained from the National Center for Biotechnology Information RefSeq database [70] on 23 February 2007. Pairwise protein BLAST [71] (blastp) was performed on this set of sequences and the expect ('e') values were arranged into a pairwise matrix. Cells with no observed protein hit were replaced with e = 1, and the diagonal was filled with e = 0. This matrix was converted to a binary matrix by assigning 1 to cells containing an e value larger than 10^-6 ^and 0 to the remaining cells, which was then hierarchically clustered in R using binary distances for generation of the distance matrix. Finally, we manually chose three illustrative groups of genes after inspection of sequences and results.

### Online search engine

Marker databases were created using the MySQL database management system, with a web interface written in PHP.

## Abbreviations

CYP, cytochrome P450 protein; Ebf, early B-cell factor; ESC, embryonic stem cell; GO, Gene Ontology; HSC, hematopoietic stem cell; mESC, murine embryonic stem cell; Nf2f, nuclear receptor subfamily 2, group F.

## Authors' contributions

PMK and MAA developed the method and prepared the manuscript.

## Additional data files

The following additional data files are available with the online version of this paper.

Additional data file 1 includes the 10,401 probe sets in the 'potential marker' set. Additional data file 2 includes the 5,848 probe sets in the 'selected marker' set. Additional data file 3 includes the 488 probe sets in 'stem cell related' set. Additional data file 4 includes identifiers of the proteins used in the phylogenetic analysis.

## Supplementary Material

Additional data file 1 Columns indicate Affymetrix probe set identifier (1 Affy id), chip set (2 chip), gene symbol (3 gene), and (4 patterns). A marker might be associated to multiple patterns, and those are encoded in column 4 using the marker server pattern identifier followed by the score for the pattern between brackets, with information for different patterns separated by a colon. Patterns can be retrieved at the marker server [21] using the marker server identifier or they can be queried with the Affymetrix probe set identifier. Click here for file

Additional data file 2 The meaning of the columns is identical to that given for Additional data file 1. Click here for file

Additional data file 3 Columns indicate Affymetrix probe set identifier (1 Affy id), chip set (2 chip), gene symbol (3 gene), and marker server pattern identifier chosen (4 pattern). Columns five and six are related to the clustering analysis (see Materials and methods): National Center for Biotechnology Information NCBI identifier for the protein sequence used (5 protein) and label for the protein cluster (6 cluster). Columns seven to 18 indicate segregation (value of 1) in pairs of stem cell samples and their differentiated derivatives, respectively: J1 mESC (7 J1-U and 8 J1-D), V6.5 mESC (9 V6.5-U and 10 V6.5-D), mast cell precursors (11 Mast-U and 12 Mast-D), mammospheres (13 MaSC-U and 14 MaSC-D), osteoblasts (15 Osteo-U and 16 Osteo-D), and hematopoietic cells (17 HSC-U and 18 HSC-D). Column 19 (multi) indicates (value 0/1) 17 genes differentially expressed along multiple stem cell lineages (one at least being non-mESC). Samples used were S255 versus S256 for mammary stem cells (undifferentiated and differentiated, respectively), S294 versus five samples (S291, S292, S293, S295, and S296) for hematopoietic stem cells (HSC), S128 versus S127 for J1 ESCs, S153 versus S175 for V6.5 ESCs, S185 versus S196 for osteoblasts, and S236 versus S237 for mast cells. Click here for file

Additional data file 4 Protein identifiers (GenBank) of the sequences used for the phylogenetic analysis depicted in Figure 5. Occasionally, the label used (for example, Ebf) differs from the gene name in the database. Labels used are derived from the phylogenetic analysis. Click here for file
