# Supplementary material for: Identification of novel stem cell markers using gap analysis of gene expression data
Source: Genome Biol. 2007 Sep 17;8(9):R193. doi: 10.1186/gb-2007-8-9-r193 (PMC2375031; doi:10.1186/gb-2007-8-9-r193)
Supplement: Additional data file 4 — Protein identifiers (GenBank) of the sequences used for the phylogenetic analysis depicted in Figure 5. Occasionally, the label used (for example, Ebf) differs from the gene name in the database. Labels used are derived from the phylogenetic analysis. [file gb-2007-8-9-r193-S4.doc]

**Additional data file 4**

**Identifiers of the proteins used in the phylogenetic analysis.** Protein identifiers (GenBank) of the sequences used for the phylogenetic analysis depicted in Figure 5. Occasionally, the label used here (e.g. Ebf) differs from the gene name in the database. Labels used here are derived from the phylogenetic analysis.

**Ebf family:**

*Strongylocentrotus purpuratus*: Ebf 115905857.

*Drosophila melanogaster:* Collier/Knot 54040796.

*Danio rerio:* Ebf2l 68438479, Ebf3 123705281, Ebf1 125836232, Ebf1l 125848606, Ebf2 122891340.

*Gallus gallus* (chicken): Ebf3 118093190, Ebf1 46048891, Ebf2 118101314.

*Canis familiaris* (dog): Ebf3 73999008, Ebf1 73953585, Ebf4 73991465, Ebf2 73993725.

*Homo sapiens* (human): Ebf3 13959320, Ebf1 31415878, Ebf4 7321167, Ebf2 113930703.

*Mus musculus* (mouse): Ebf3 44890374, Ebf1 56205836, Ebf4 26006701, Ebf2 3757778.

*Xenopus laevis* (frog): Ebf3 13959679, Ebf2 2773361.

*Tetraodon nigroviridis*: Ebf3 47222306, Ebf1 47221809, Ebf2 47226451.

*Monodelphis domestica* (opossum): Ebf1 126291374, Ebf3 126332020, Ebf2 126305447

**Rab3 family:**

*Xenopus laevis* (frog): rab3a 54311426, rab3 52139114.

*Xenopus tropicalis*: rab3a 62858351, rab3b 134023761.

*Monodelphis domestica* (opossum): rab3a 126323469.

*Canis familiaris* (dog): rab3a 73986147, rab3c 73949766, rab3b 73976938, rab3d 57101400.

*Bos taurus* (cow): rab3a 110350677, rab3c 28875789, rab3b 27806113.

*Mus musculus* (mouse): rab3a 6679593, rab3c 13470090, rab3b 12963723, rab3d 15042957.

*Danio rerio*: rab3a (2) 62955495, rab3a (2) 51010929, rab3c? 50540122, rab3b 68399356, rab3d (2) 125804524, rab3d (2) 50540426.

*Tetraodon nigroviridis*: rab3a (2) 47217415, rab3a (2) 47216418, rab3c? 47217500, rab3d 47212241.

*Monodelphis domestica* (opossum): rab3c 126315213, rab3b 126305656, rab3d 126322833.

*Strongylocentrotus purpuratus*: rab3 115681598.

*Drosophila melanogaster*: rab3 17737457.

*Gallus gallus* (chicken): rab3b 118094547.

**Cyp1a/b family:**

*Mus musculus* (mouse): cyp1b1 6753568, cyp1a2 6753566, cyp1a1 6753564.

*Strongylocentrotus purpuratus*: cyp1a/b (4) 115946034, cyp1a/b (4) 115946028, cyp1a/b (4) 115956490, cyp1a/b (4) 115679730.

*Danio rerio*: cyp1a (2) 55249637, cyp1a (2) 40538770, cyp1b1 (2) 57547558, cyp1b1 (2) 68421589.

Monodelphis domestica (opossum): cyp1a? 126334778, cyp1a1 126272482, cyp1a2 126272484, cyp1b1 126304566.

*Tetraodon nigroviridis*: cyp1a 47220920, cyp1b1 47230274.

*Xenopus laevis* (frog): cyp1a 4140246.

*Phalacrocorax carbo* (cormorant): cyp1a (2) 93277049, cyp1a (2) 93277051.

*Gallus gallus* (chicken): cyp1a (2) 1783318, cyp1a (2) 45384068.

*Bos taurus* (cow): cyp1a1 21361058, cyp1a2 119913810, cyp1b1 61887409.

*Canis familiaris* (dog): cyp1a1 3913305, cyp1a2 59958379, cyp1b1 73980746.

*Homo sapiens* (human): cyp1a1 4503199, cyp1a2 73915100, cyp1b1 21389156.

*Drosophila melanogaster*: NONE

**nr2f1 family:**

*Strongylocentrotus purpuratus*: COUP 115916057.

*Tetraodon nigroviridis*: nr2f1 47211605, nr2f2 47224170.

*Danio rerio*: nr2f1 20589472, nr2f1l 55925486, nr2f2 24111246.

*Homo sapiens* (human): nr2f1 5032173, nr2f2 14149746.

*Mus musculus* (mouse): nr2f1 111185902, nr2f2 73611910.

*Xenopus laevis* (frog): nr2f1 5353742, nr2f2 27924187.

*Scyliorhinus canicula*: nr2f1 44969507.

*Petromyzon marinus*: nr2f 28630300.

*Drosophila melanogaster*: svp 17737921.

*Ciona intestinalis*: COUP 118343705.

*Branchiostoma floridae*: COUP 28974300.
